# Impact of interstitial lung disease on left ventricular myocardial function

**DOI:** 10.1371/journal.pone.0286423

**Published:** 2024-02-06

**Authors:** Max Jonathan Stumpf, Marina Michaela Luise Wirtz, Max Fabian Fleddermann, Leonie Biener, Leonie Weinhold, Marcel Weber, Christian Alexander Schaefer, Georg Nickenig, Dirk Skowasch, Carmen Pizarro

**Affiliations:** 1 Department of Internal Medicine II - Pneumology/Cardiology, University Hospital Bonn, Bonn, Germany; 2 Institute for Medical Biometry, Informatics and Epidemiology, University Hospital Bonn, Bonn, Germany; Scuola Superiore Sant’Anna, ITALY

## Abstract

**Background:**

Interstitial lung disease (ILD) comprises a wide variety of pulmonary parenchymal disorders within which progressive fibrosing ILD (PF-ILD) constitutes a phenotypic subset. By use of speckle tracking-based strain analysis we aimed to evaluate the degree of left ventricular (LV) dysfunction in progressive vs. non-progressive fibrosing ILD (non-PF-ILD).

**Methods:**

A total of 99 ILD patients (mean age 63.7 ± 13.5 years, 37.4% female), composed of 50 PF-ILD and 49 non-PF-ILD patients, and 33 controls were prospectively enrolled and underwent conventional and speckle tracking echocardiography. Additional laboratory and pulmonary function testing, as well as six-minute walk test were performed.

**Results:**

As compared to the non-PF-ILD cohort, PF-ILD patients exhibited a significantly impaired forced vital capacity (2.4 ± 1.0l vs. 3.1 ± 0.9l, p = 0.002), diffusion capacity for carbon monoxide (DL_CO_, 25.6 ± 16.3% predicted vs. 43.6 ± 16.67% predicted, p <0.001) and exercise capacity response as measured by the six-minute walk test distance (268.1 ± 178.2m vs. 432.6 ± 94.2m, p <0.001). Contrary to conventional echocardiographic LV parameters, both regional and global longitudinal LV strain measurements were significantly altered in ILD patients as compared to controls. No differences in LV strain were found between both patient groups. Significant correlations were observed between global longitudinal strain, on the one hand, and systemic inflammation markers, total lung capacity (TLC) and DL_CO_, on the other hand (high-sensitivity C-reactive protein: Pearson´s r = -0.30, p< 0.001; interleukin-6: Pearson´s r = -0.26, p = 0.007; TLC % predicted: Pearson´s r = 0.22, p = 0.02; DL_CO_ % predicted: Pearson´s r = 0.21, p = 0.02).

**Conclusions:**

ILD is accompanied by LV dysfunction. LV functionality inversely correlates with the severity of the restrictive ventilatory defect and inflammation marker levels. These observations support the assumption of persistent low-grade systemic inflammation that may link systemic cardiovascular function to ILD status.

## Introduction

Interstitial lung disease (ILD) encompasses a large group of more than 200 parenchymal lung disorders that share similar clinical, radiological and pathophysiological characteristics [1]. A proportion of ILD patients may develop a progressive fibrotic phenotype. A consented definition on progressive fibrosing ILD (PF-ILD) was provided by the INBUILD study [2]. Progression was defined by clearly specified combinations of worsening in pulmonary function, respiratory symptomatology and high-resolution computed tomography (HRCT) over the preceding 24 months despite treatment. PF-ILDs comprise a broad range of underlying types of ILD. Apart from idiopathic pulmonary fibrosis (IPF)–the archetypal PF-ILD–autoimmune ILD, chronic hypersensitivity pneumonitis, idiopathic non-specific interstitial pneumonia (NSIP) or chronic sarcoidosis, inter alia, may exhibit a progressive fibrosing course. Regardless of ILD´s aetiology, PF-ILD show similarities in the pathogenetic mechanisms leading to self-sustaining fibrosis [3].

Progressive fibrosis substantially impacts morbidity and mortality. Morbidity, in turn, is often amplified by pulmonary and extrapulmonary comorbidities. In IPF, cardiac comorbidities constitute the second most common cause of death, accounting for approximately 10% of deaths, only surpassed by respiratory failure [4]. Ischaemic heart disease, in turn, represents the most frequent cardiovascular comorbid condition in ILD with a prevalence up to 68% [5]. Available evidence suggests that cardiovascular morbidity in IPF arises not only from shared risk factors, but from a systemic proinflammatory state. Systemic cytokine derangements in IPF support this notion [6]. It is noteworthy that even fibrotic lung diseases other than IPF are accompanied with an increased prevalence of ischaemic heart disease, even after adjustment for common risk factors [7].

For the detection of early left ventricular (LV) structural abnormalities preceding overt left heart diseases, two-dimensional speckle tracking echocardiography represents a sensitive imaging technique. It permits visualization of subtle disturbances of LV myocardial deformation properties and has thus proved superior to conventional echocardiography [8].

In keeping with this, the aim of this prospective cohort study was: 1) to evaluate LV functionality–assessed by two-dimensional speckle tracking echocardiography–as a function of progressive fibrosing versus non-progressive fibrosing ILD, and 2) to compare the results with those obtained in healthy controls.

## Materials and methods

### Study population

Between December 2018 and November 2020, a total of 104 patients aged ≥ 18 years receiving treatment for ILD at the outpatient Department of Pneumology, University Hospital Bonn (Bonn, Germany), were screened for this prospective cohort study. Five patients were excluded due to either acute exacerbated ILD at the time of evaluation or their denial to undergo cardiac diagnostics. ILD- diagnosis relied on consensus by multidisciplinary discussion. Differentiation between PF-ILD and non-PF-ILD was based on the INBUILD study criteria [2]. PF-ILD was defined by at least one of the following criteria, fulfilled within 24 months, despite standard treatment: a) relative decline in forced vital capacity (FVC) ≥ 10% predicted, b) relative decline in FVC ≥ 5–10% predicted and worsened respiratory or increased extent of fibrosis on HRCT, or c) worsened respiratory symptoms and increased extent of fibrosis on HRCT. Those ILD patients who did not fulfil the INBUILD criteria were referred to as non-PF-ILD. In addition to the ILD patient cohort, 33 healthy controls without lung function abnormalities were included. Controls were matched against the entire ILD cohort for age, gender and cardiovascular risk factors. ILD patients and controls underwent pulmonary function testing and capillary blood gas analysis, laboratory testing, exercise capacity testing by means of the six-minute walk test (6-MWT) and evaluation of LV myocardial contractility by transthoracic echocardiographic speckle tracking analysis. Written informed consent was obtained from each participant. The study was performed in line with the principles of the 1975 Declaration of Helsinki. Approval was obtained from the Medical Ethics Committee of the University of Bonn (Germany).

### Pulmonary function testing and capillary blood gas analysis

Pulmonary function testing was performed in conformity with the European Respiratory Society guidelines [9] and comprised spirometry, bodyplethysmography and determination of diffusion capacity for carbon monoxide. Static and dynamic lung volumes, such as forced expiratory volume in one second (FEV1), Tiffeneau-index (FEV1/VC), FVC, total lung capacity (TLC) and diffusion capacity for carbon monoxide (DL_CO_) as assessed by the single-breath technique were measured. Parameters were recorded as absolute measures and as percentages of the predicted values (Bodyplethismograph Jaeger©, Alveo-Diffusionstest Jaeger©, Wuppertal, Germany).

Capillary blood gas analysis was performed in rest and after exercise (6MWT).

### Six-minute walk test

Physical performance was assessed by 6-MWT, performed to ATS standards [10]. 6-MWT was carried out indoors, along a long, flat corridor with a hard surface. The walking course was 30 m in length. Participants were instructed to walk as fast as possible. Measurements comprised walk distance as well as pre- and post-exercise heart rate, breathing rate and Borg dyspnoea and exhaustion score. The higher the score, the higher the perceived dyspnoea and exhaustion, respectively.

### Laboratory testing

Blood samples were acquired to assess full blood count, N-terminal pro–brain natriuretic peptide (NT-proBNP) levels, HbA1c concentration and high-sensitivity C-reactive protein (hs-CRP). Interleukin-6 (IL-6) levels, a proinflammatory cytokine reported to impact fibrotic response, were measured. Additionally, a complete lipid panel with lipids and lipoproteins that served as cardiovascular risk stratifiers was assessed.

### Transthoracic echocardiography and speckle tracking analysis

Each study participant underwent a complete standardized transthoracic echocardiography, performed by experienced cardiac sonographers at the University Hospital´s Department of Cardiology. Echocardiographic studies were conducted in line with the current recommendations of the European Association of Cardiovascular Imaging [11], using a 2.5 MHz phased-array transducer and a commercially available ultrasound system (iE33, Philips Medical Systems, Andover, Massachusetts; GE Vivid E9, GE Health Medical, Horten, Norway). With the patient lying in the left lateral position, parasternal and apical view parameters were obtained. In supine position, subcostal view measurements were recorded.

Two-dimensional speckle tracking analysis was performed offline after digitalization and transfer of the echocardiographic data set to a dedicated workstation (TomTec Image Arena, Munich, Germany). The LV endocardium was manually traced and tracked by software throughout two cardiac cycles. LV myocardial performance was objectified by assessment of longitudinal strain. Regional longitudinal strain was obtained by apical, midventricular and basal segmentation of the LV. Global longitudinal strain derived as the average of the regional strain values. The lower the strain values, the better the myocardial contractility.

### Statistical analysis

Continuous variables are expressed as mean ± standard deviation. Categorial parameters are presented as absolute number and percentage. Student´s *t*-test for independent variables and univariate analysis of variance was used for comparison of continuous data between groups, as appropriate. For comparison of categorical parameters, Pearson’s chi-squared test was employed. Correlation was assessed by Pearson´s correlation coefficient and Cramer´s V, in case of nominal scaled variables. As the evaluation of LV functionality comprised seven strain values (global longitudinal strain and six segmental strain indices), a p-value < 0.0072, according to Bonferroni correction for multiple testing, was considered statistically significant for strain analysis. For the remaining measurements, statistical significance was assumed when the null hypothesis could be rejected at p < 0.05. Intergroup differences regarding LV strain measures were assessed by linear regression models adjusted for age to account for this confounder. All statistical analyses were performed using SPSS Statistics version 26.0 (IBM, Armonk, NY, USA) and R version 4.0.3 software.

## Results

### Characteristics of the study population

Demographic characteristics and clinical data of the study population are given in Table 1. 50 out of 99 ILD patients met the PF-ILD criteria, 49 patients exhibited a non-PF-ILD. The underlying ILD aetiology differed significantly between both patient groups. Within the PF-ILD cohort, IPF was present in half of the cases (n = 25, 50.0%), followed by ILD due to autoimmune diseases (n = 9, 18.0%). In non-PF-ILD, stage IV sarcoidosis and autoimmune ILD equally constituted the most common underlying ILD types, each with n = 15 (30.6%).

**Table 1:** Demographics and clinical characteristics.

	PF-ILD (n = 50)	Non-PF-ILD (n = 49)	All ILD patients (n = 99)	Controls (n = 33)	P-value* (PF-ILD vs. controls)	P-value** (non-PF-ILD vs. controls)	P-value*** (PF-ILD vs. non-PF-ILD)
**Demographics**							
Female	17 (34.0%)	20 (40.8%)	37 (37.4%)	20 (60.6%)	n.s.	n.s.	n.s.
Age [years]	68.6 ± 10.7	58.8 ± 14.2	63.7 ± 13.5	62.5 ± 10.4	0.01	n.s.	<0.001
**Cardiovascular risk factors**							
Arterial hypertension	24 (48.0%)	21 (42.9%)	45 (45.5%)	16 (48.5%)	n.s.	n.s.	n.s.
Dyslipidaemia	18 (36.0%)	21 (42.9%)	39 (39.4%)	9 (27.3%)	n.s.	n.s.	n.s.
Diabetes mellitus	12 (24.0%)	9 (18.4%)	21 (21.2%)	3 (9.1%)	n.s.	n.s.	n.s.
BMI [kg/m^2^]	26.1 ± 5.1	27.0 ± 5.7	26.5 ± 5.3	27.0 ± 4.8	n.s.	n.s.	n.s.
Packyears	16.7 ± 20.8	11.3 ± 19.2	14.1 ± 20.1	12.7 ± 23.6	n.s.	n.s.	n.s.
Chronic kidney disease	8 (16.0%)	6 (12.2%)	14 (14.1%)	1 (3.0%)	n.s.	n.s.	n.s.
**Medication**							
Platelet inhibitor	19 (38.0%)	10 (20.4%)	29 (29.3%)	6 (18.2%)	n.s.	n.s.	n.s.
RAAS-Inhibitor	16 (32.0%)	15 (30.6%)	31 (31.3%)	9 (27.3%)	n.s.	n.s.	n.s.
ß-Blocker	21 (42.0%)	15 (30.6%)	36 (36.4%)	8 (24.2%)	n.s.	n.s.	n.s.
Statin	30 (60.0%)	20 (40.8%)	50 (50.5%)	6 (18.2%)	0.001	n.s.	n.s.
Calcium antagonist	8 (16.0%)	3 (6.1%)	11 (11.1%)	3 (9.1%)	n.s.	n.s.	n.s.
Nintedanib	18 (36.0%)	0 (0%)	18 (18.2%)	0 (0%)	N/A	N/A	< 0.001
Pirfenidone	8 (16.0%)	0 (0%)	8 (8.1%)	0 (0%)	N/A	N/A	<0.001
Inhaled glucocorticoid	12 (24.0%)	10 (20.4%)	22 (22.2%)	0 (0%)	n.s.	n.s.	n.s.
Systemic glucocorticoid	28 (56.0%)	34 (69.4%)	62 (62.6%)	1 (3.0%)	<0.001	<0.001	n.s.
**ILD aetiology**					N/A	N/A	<0.001
IPF	25 (50.0%)	0 (0%)	25 (25.3%)	0 (0%)			
Stage IV sarcoidosis	2 (4.0%)	15 (30.6%)	17 (17.2%)	0 (0%)			
Autoimmune disease	9 (18.0%)	15 (30.6%)	24 (24.2%)	0 (0%)			
iNSIP	6 (12.0%)	5 (4.1%)	11 (11.1%)	0 (0%)			
Exposure related	2 (4.0%)	2 (4.1%)	4 (4.0%)	0 (0%)			
CPFE	3 (6.0%)	4 (8.2%)	7 (7.1%)	0 (0%)			
Chronic hypersensitivity pneumonitis	1 (2.0%)	1 (2.0%)	2 (2.0%)	0 (0%)			
Unclassified IIPs	2 (4.0%)	7 (14.3%)	9 (9.1%)	0 (0%)			

Data are presented as n (%) or mean ± standard deviation.

*P-value refers to data comparison between the PF-ILD patient group and controls.

**P-value refers to data comparison between the non-PF-ILD patient group and controls.

*** P-value refers to data comparison between the PF-ILD and the non-PF-ILD patient group.

P-values are significant at <0.05.

Abbreviations:

BMI: Body mass index; CPFE: Combined pulmonary fibrosis and emphysema; IIPs: Idiopathic interstitial pneumonias; IPF: Idiopathic pulmonary fibrosis; N/A: Not applicable; non-PF-ILD: Non-progressive fibrosing interstitial lung disease; iNSIP: Idiopathic nonspecific interstitial pneumonia; PF-ILD: Progressive fibrosing interstitial lung disease.

The mean age did not substantially differ between the entire ILD cohort and controls (63.7 ± 13.5 years vs. 62.5 ± 10.4 years, respectively; p = 0.62). However, PF-ILD patients were significantly older than non-PF-ILD patients (68.6 ± 10.8 years vs. 58.8 ± 14.2 years, respectively; p <0.001) and controls (p = 0.01). Prior or continued nicotine consumption was present in n = 31/50 (62.0%) and n = 23/49 (39.0%) of PF-ILD and non-PF-ILD patients, respectively. The number of packyears did not significantly differ neither between both patient groups (p = 0.18) nor as compared to controls (p = 0.41 for PF-ILD vs. controls; p = 0.78 for non-PF-ILD vs. controls). Likewise, no significant intercohortal differences were detected for other cardiovascular risk factors beyond smoking.

### Pulmonary function testing and six-minute walk test

**Fig 1:**
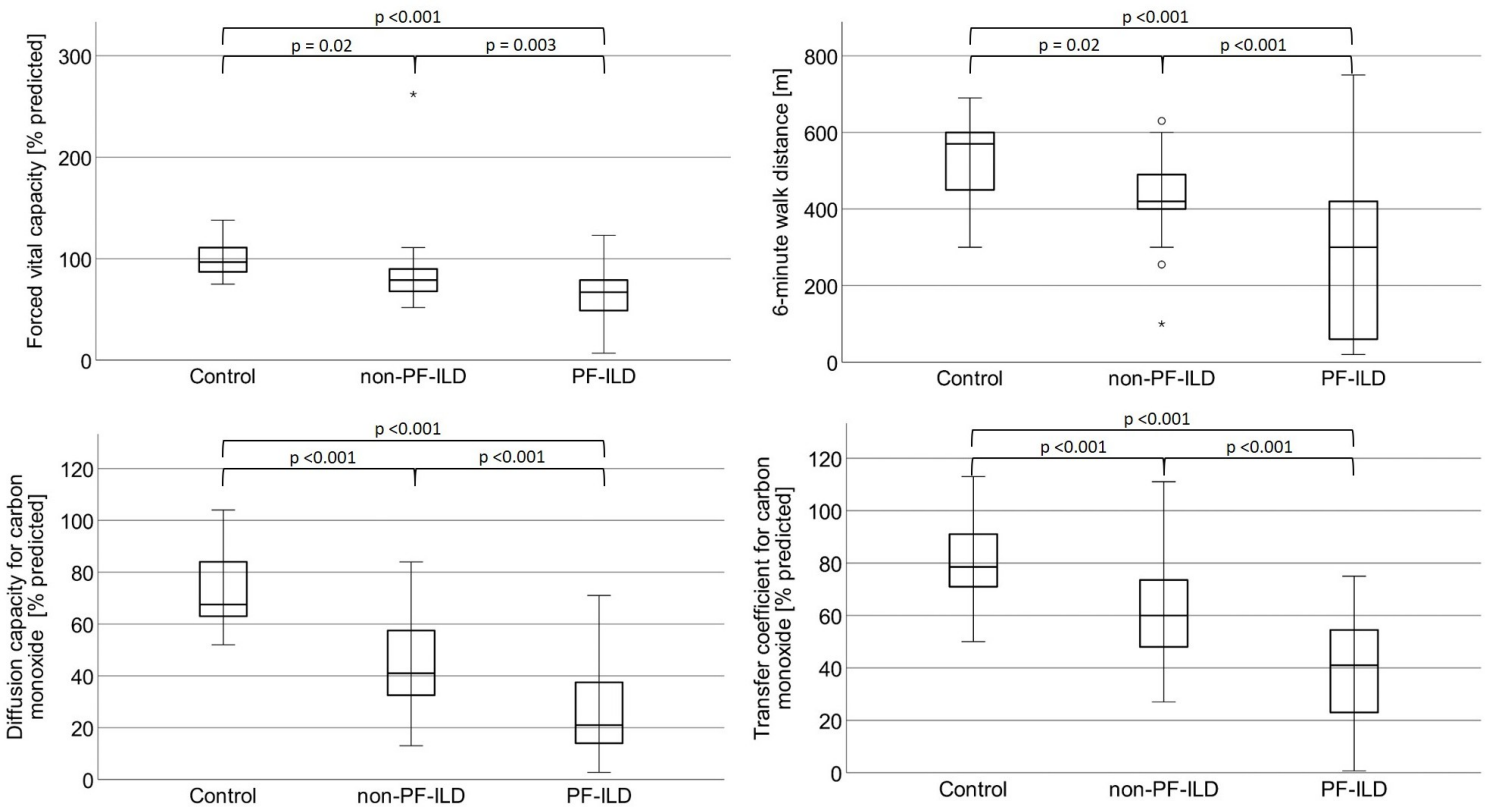
Results obtained by pulmonary function testing and six-minute walk test. Abbreviations: Non-PF-ILD: Non-progressive fibrosing interstitial lung disease; PF-ILD: Progressive fibrosing interstitial lung disease.

Pulmonary function testing revealed a FVC of 2.4 ± 1.1l in absolute terms in the PF-ILD group that was significantly lower than the FVC offered by non-PF-ILD patients (3.1 ± 0.9l; p = 0.002). The same holds true for the transfer factor and the transfer coefficient, as summarized in Fig 1 and Table 2. However, TLC in absolute terms did not significantly differ between both patient groups. 14 patients with PF-ILD (36.8%) and two patients with non-PF-ILD (4.4%; p<0.001) were under long term supplemental oxygen therapy.

**Table 2:** Results obtained by pulmonary function testing and six-minute walk test.

	PF-ILD (n = 50)	Non-PF-ILD (n = 49)	All ILD patients (n = 99)	Controls (n = 33)	P-value* (PF-ILD vs. controls)	P-value** (non-PF-ILD vs. controls)	P-value*** (PF-ILD vs. non-PF-ILD)
**Pulmonary function parameters**							
TLC [L]	4.9 ± 1.8	5.4 ± 1.3	5.1 ± 1.6	6.3 ± 1.2	<0.001	0.003	n.s.
TLC [% predicted]	76.9 ± 28.3	86.7 ± 15.4	81.8 ± 23.2	104.4 ± 16.0	<0.001	<0.001	0.04
FVC [L]	2.4 ± 1.0	3.1 ± 0.9	2.8 ± 1.0	3.6 ± 0.9	<0.001	0.01	0.002
FVC [% predicted]	65.9 ± 23.3	82.8 ± 30.6	74.3 ± 28.4	99.0 ± 16.7	<0.001	0.02	0.003
FEV1 [L]	2.0 ± 0.8	2.4 ± 0.7	2.2 ± 0.8	3.0 ± 0.7	<0.001	0.001	n.s.
FEV_1_ [% predicted]	68.6 ± 24.3	75.6 ± 17.5	72.1 ± 21.4	99.1 ± 14.3	<0.001	<0.001	n.s.
FEV_1_/VC [%]	84.7 ± 10.9	80.9 ± 7.29	82.8 ± 9.4	84.7 ± 5.7	n.s.	0.02	0.04
DL_CO_ [% predicted]	25.6 ± 16.3	43.6 ± 16.67	35.5 ± 18.7	72.3 ± 13.3	< 0.001	< 0.001	<0.001
DL_CO_/VA [% predicted]	39.2 ± 20.9	60.6 ± 19.6	50.9 ± 22.7	80.0 ± 16.2	< 0.001	<0.001	< 0.001
LTOT	14 (28.0%)	2 (4.1%)	16 (16.2%)	0 (0%)	< 0.001	n.s.	<0.001
O_2_-Flow [L/min]	1.1 ± 1.7	0.1 ± 0.7	0.56 ± 1.3	0	0.001	n.s.	0.001
**Six-minute walk test**							
Pre-6MWT examination							
Heart rate [1/min]	74.7 ± 16.1	81.7 ± 13.66	78.5 ± 15.1	71.7 ± 11.2	n.s.	0.004	0.04
Breathing rate [1/min]	20.0 ± 5.0	18.0 ± 3.0	19.3 ± 3.9	15.0 ± 4.0	<0.001	<0.001	n.s.
Borg-Scale: Dyspnoea	1.8 ± 1.7	1.4 ± 1.5	1.6 ± 1.6	0.5 ± 0.7	0.001	0.008	n.s.
Borg-Scale: Exhaustion	2.3 ± 2.3	1.5 ± 1.7	1.9 ± 2.0	0.9 ± 2.0	0.02	n.s.	n.s.
pO_2_ [mmHg]	69.5 ± 13.4	76.0 ± 11.6	72.9 ± 12.8	79.8 ± 8.8	0.002	n.s.	0.02
pCO_2_ [mmHg]	34.2 ± 4.8	33.6 ± 4.2	33.9 ± 4.56	34.0 ± 3.5	n.s.	n.s.	n.s.
sO_2_ [%]	93.3 ± 6.3	95.7 ± 2.5	94.6 ± 4.8	96.7 ± 1.7	0.02	n.s.	0.04
pH	7.45 ± 0.04	7.45 ± 0.04	7.45 ± 0.04	7.45 ± 0.04	n.s.	n.s.	n.s.
**6-MWT distance [m]**	268.1 ± 178.2	432.6 ± 94.2	348.6 ± 164.9	522.4 ± 108.8	< 0.001	0.02	< 0.001
Post-6MWT examination							
Heart rate [1/min]	89.9 ± 18.7	101.3 ± 16.7	96.3 ± 18.4	94.0 ± 18.8	n.s.	n.s.	0.008
Breathing rate [1/min]	27.2 ± 7.8	24.2 ± 4.9	25.5 ± 6.5	19.9 ± 3.3	<0.001	<0.001	n.s.
Borg-Scale: Dyspnoea	4.4 ± 1.6	3.1 ± 2.3	3.7 ± 2.1	1.1 ± 0.9	< 0.001	<0.001	0.008
Borg-Scale: Exhaustion	3.8 ± 2.1	2.9 ± 2.3	3.3 ± 2.3	1.2 ± 1.9	0.001	<0.01	n.s.
pO_2_ [mmHg]	71.4 ± 21.6	76.5 ± 13.2	74.2 ± 17.5	90.7 ± 8.4	0.001	<0.001	n.s.
pCO_2_ [mmHg]	33.7 ± 5.1	35.1 ± 4.7	34.5 ± 4.9	33.9 ± 3.4	n.s.	n.s.	n.s.
sO_2_ [%]	89.0 ± 8.6	92.4 ± 8.9	91.0 ± 8.9	96.8 ± 2.0	0.001	0.02	n.s.
pH	7.44 ± 0.04	7.43 ± 0.04	7.43 ± 0.04	7.44 ± 0.04	n.s.	n.s.	n.s.

Data are presented as n (%) or mean ± standard deviation.

*P-value refers to data comparison between the PF-ILD patient group and controls.

**P-value refers to data comparison between the non-PF-ILD patient group and controls.

*** P-value refers to data comparison between the PF-ILD and the non-PF-ILD patient group.

P-values are significant at <0.05.

Abbreviations: DL_CO_: Diffusion capacity of the lung for carbon monoxide; FEV1: Forced expiratory volume in 1 s; FVC: Forced vital capacity; LTOT: Long term oxygen therapy; non-PF-ILD: Non-progressive fibrosing interstitial lung disease; PF-ILD: Progressive fibrosing interstitial lung disease; PCO2: Carbon dioxide tension; PO2: Oxygen tension; sO2: Oxygen saturation; VA: Alveolar volume; 6-MWT: Six-minute walk test.

As compared to non-PF-ILD patients, PF-ILD was associated with a substantially reduced exercise response, measured by 6-MWT distance (268 ± 178m vs. 433 ± 94m; p <0.001), that was accompanied by a higher amount of post-exercise dyspnoea according to the Borg scale (p = 0.008).

### Laboratory testing

**Fig 2:**
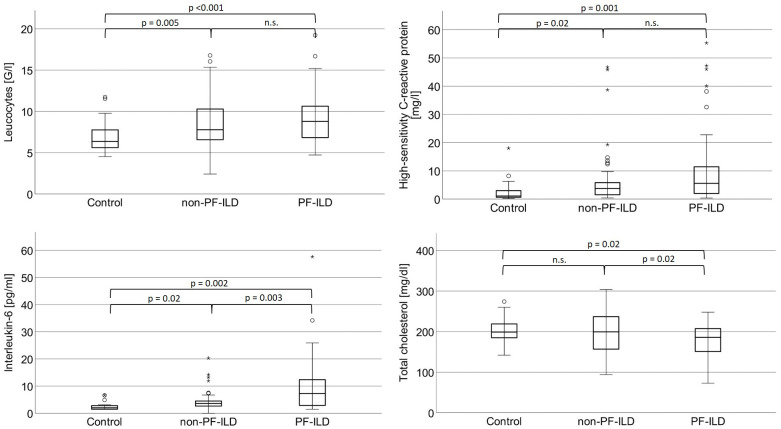
Results obtained by laboratory testing. Abbreviations: Non-PF-ILD: Non-progressive fibrosing interstitial lung disease; n.s.: Not significant; PF-ILD: Progressive fibrosing interstitial lung disease.

Laboratory testing evidenced elevated IL-6 levels among PF-ILD patients as compared to non-PF-ILD patients (9.7 ± 10.3 pg/ml vs. 4.56 ± 3.8 pg/ml; p = 0.003) and controls (6.8 ± 1.8 pg/ml; p = 0.002; Fig 2). White blood cell counts revealed lower leukocytes in the control group than in the patient cohorts (p<0.001 for PF-ILD patients and p = 0.005 for non-PF-ILD patients). The same applies to hs-CRP levels, as given in Table 3. All three here studied markers of systemic inflammation (total leukocyte count, hs-CRP and IL-6) were significantly associated with pulmonary testing parameters indicating restrictive ventilatory limitation (TLC, FVC) or diffusion impairment (DL_CO_, DL_CO_/alveolar volume).

**Table 3:** Results obtained by laboratory testing.

	PF-ILD (n = 50)	Non-PF-ILD (n = 49)	All ILD patients (n = 99)	Controls (n = 33)	P-value* (PF-ILD vs. controls)	P-value*** (non-PF-ILD vs. controls)	P-value*** (PF-ILD vs. non-PF-ILD)
Leucocytes [10^9^/ml]	9.1 ± 3.0	8.6 ± 3.0	8.8 ± 3.0	6.8 ± 1.8	<0.001	0.005	n.s.
Haemoglobin [g/dl]	14.0 ± 2.8	13.9 ± 1.9	14.0 ± 2.4	14.1 ± 1.1	n.s.	n.s.	n.s.
Thrombocytes [10^9^/ml]	234.6 ± 81.7	243.7 ± 85.0	239.2 ± 83.0	239.9 ± 79.1	n.s.	n.s.	n.s.
HbA_1c_ [%]	6.0 ± 0.9	5.8 ± 0.9	5.9 ± 0.9	5.6 ± 0.6	n.s.	n.s.	n.s.
Triglycerides [mg/dl]	161.7 ± 82.3	209.0 ± 205.0	185.1 ± 156.6	186.3 ± 117.9	n.s.	n.s.	n.s.
Total cholesterol [mg/dl]	179.0 ± 34.0	200.5 ± 48.3	189.5 ± 45.3	203.1 ± 34.8	0.02	n.s.	0.02
LDL cholesterol [mg/dl]	113.4 ± 36.2	123.4 ± 40.6	118.4 ± 38.6	129.7 ± 34.0	n.s.	n.s.	n.s.
HDL cholesterol [mg/dl]	56.6 ± 22.7	55.5 ± 20.6	56.0 ± 21.3	56.7 ± 16.1	n.s.	n.s.	n.s.
Lipoprotein(a) [nmol/l]	66.2 ± 111.6	46.3 ± 65.3	56.1 ± 91.3	27.3 ± 48.7	n.s.	n.s.	n.s.
Hs-CRP [mg/l]	10.7 ±13.4	6.9 ± 10.4	8.8 ± 12.1	2.4 ± 3.5	0.001	0.02	n.s.
Interleukin-6 [pg/ml]	9.7 ± 10.3	4.6 ± 3.8	7.1 ± 8.1	2.5 ± 1.6	0.002	0.02	0.003
NT-proBNP [ng/l]	267.1 ± 228.4	191.6 ± 186.7	227.7 ± 209.9	84.2 ± 55.3	<0.001	0.003	n.s.

Data are presented as mean ± standard deviation.

*P-value refers to data comparison between the PF-ILD patient group and controls.

**P-value refers to data comparison between the non-PF-ILD patient group and controls.

*** P-value refers to data comparison between the PF-ILD and the non-PF-ILD patient group.

Unpaired *t*-test was applied. P-values are significant at <0.05.

Abbreviations: HDL: High-density lipoprotein; Hs-CRP: High-sensitivity C-reactive protein; LDL: Low-density lipoprotein; non-PF-ILD: Non-progressive fibrosing interstitial lung disease; NT-proBNP: N-terminal prohormone of brain natriuretic peptide; PF-ILD: Progressive fibrosing interstitial lung disease.

### Conventional and speckle tracking echocardiography

**Fig 3:**
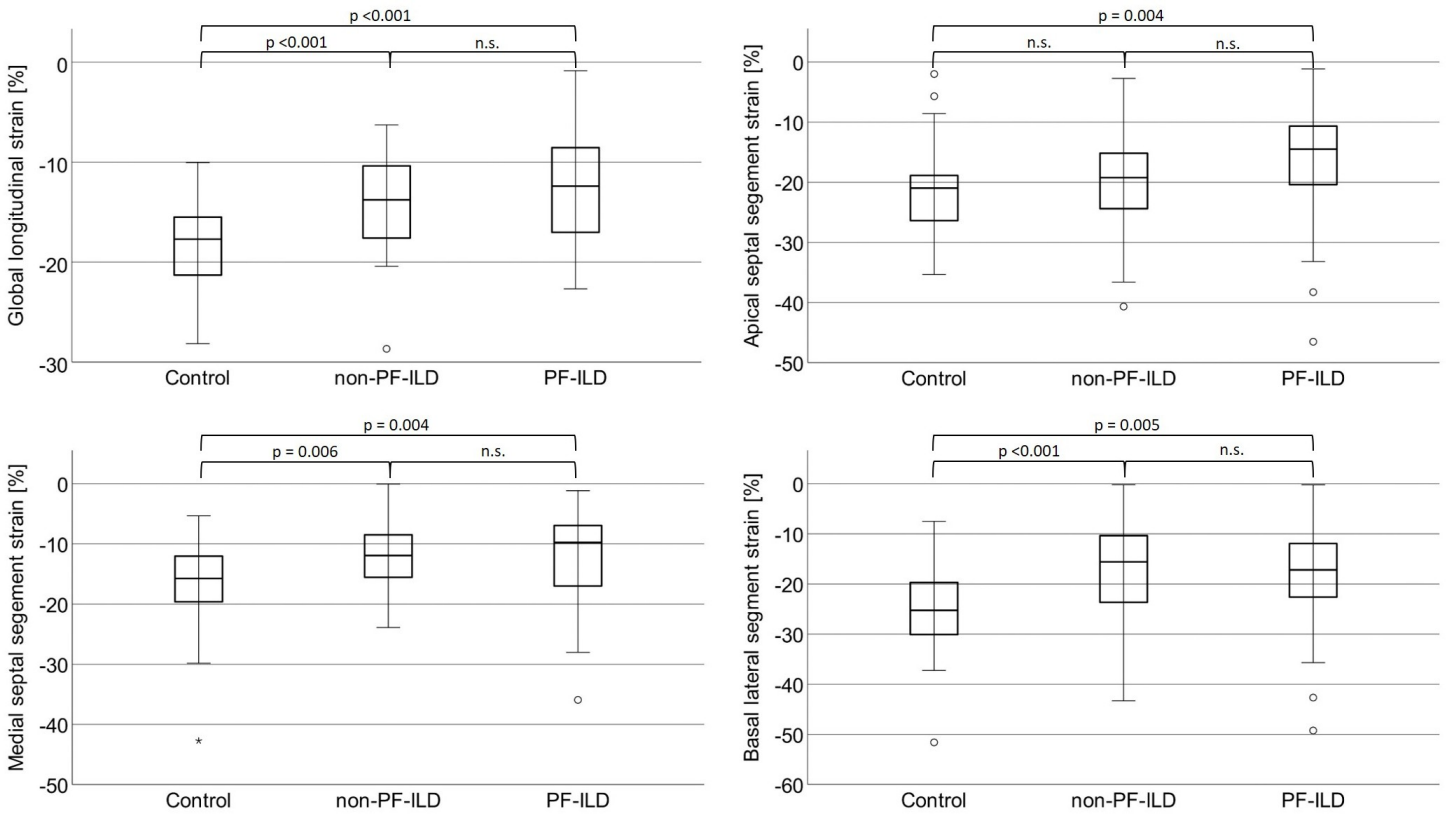
Results obtained by left ventricular speckle tracking-based echocardiography. Abbreviations: Non-PF-ILD: Non-progressive fibrosing interstitial lung disease; n.s.: Not significant; PF-ILD: Progressive fibrosing interstitial lung disease.

Conventional echocardiography showed an overall preserved systolic LV function with no differences between groups, as presented in Table 4. The same holds true for diastolic LV function that did not significantly differ between groups. In contrast to conventional echocardiographic parameters, speckle tracking based longitudinal strain analysis evidenced significantly impaired global and regional LV deformation properties in ILD patients as compared to controls (Fig 3).

**Table 4:** Results obtained by conventional and speckle tracking-based echocardiography.

	PF-ILD (n = 50)	Non-PF-ILD (n = 49)	All ILD patients (n = 99)	Controls (n = 33)	P-value* (PF-ILD vs. controls)	P-value** (non-PF-ILD vs. controls)	P-value*** (PF-ILD vs. non-PF-ILD)
**Conventional echocardiography** ^1^							
Left ventricular ejection fraction [%]	59.1 ± 8.4	60.7 ± 5.2	59.9 ± 7.0	61.0 ± 3.9	n.s.	n.s.	n.s.
Diastolic dysfunction ≥I°	29 (58.0%)	26 (53.1%)	55 (55.6%)	17 (51.5%)	n.s.	n.s.	n.s.
Aortic valve disease	17 (34.0%)	15 (30.6%)	32 (32.3%)	5 (15.2%)	n.s.	n.s.	n.s.
Mitral valve disease	44 (88.0%)	41 (83.7%)	85 (85.9%)	23 (69.7%)	n.s.	n.s.	n.s.
Systolic pulmonary artery pressure [mmHg]	42.7 ± 21.1	34.1 ± 20.3	39.1 ± 21.0	27.2 ± 6.5	n.s.	n.s.	n.s.
TAPSE [cm]	2.1 ± 0.5	2.3 ± 0.5	2.2 ± 0.5	2.4 ± 0.4	0.002	n.s.	0.01
**Speckle tracking echocardiography, left ventricular longitudinal strain assessment** ^2^							
Global strain [%]	-12.7 ± 5.5	-14.1 ± 4.6	-13.4 ± 5.2	-18.3 ± 4.2	<0.001	<0.001	n.s.
Apical septal [%]	-16.2 ± 9.2	-19.9 ± 8.3	-18.1 ± 8.9	-21.6 ± 7.4	0.004	n.s.	n.s.
Medial septal [%]	-11.5 ± 7.6	-12.0 ± 5.3	-11.8 ± 6.5	-16.4 ± 7.5	0.004	0.006	n.s.
Basal septal [%]	-10.7 ± 6.4	-11.4 ± 7.9	-11.1 ± 7.2	-15.4 ± 8.3	n.s.	n.s.	n.s.
Apical lateral [%]	-8.4 ± 8.3	-9.9 ± 7.6	-9.1 ± 7.9	-12.2 ± 7.1	n.s.	n.s.	n.s.
Medial lateral [%]	-13.5 ± 7.6	-14.2 ± 10.0	-13.9 ± 8.8	-21.0 ± 9.4	0.006	0.003	n.s.
Basal lateral [%]	-18.0 ±9.8	-17.2 ± 10.5	-17.6 ± 10.1	-25.5 ± 9.1	0.005	0.001	n.s.

Data are presented as n (%) or mean ± standard deviation.

*P-value refers to data comparison between the PF-ILD patient group and controls.

**P-value refers to data comparison between the non-PF-ILD patient group and controls.

*** P-value refers to data comparison between the PF-ILD and the non-PF-ILD patient group.

^1^ Intergroup differences calculated via unpaired *t*-test. P-values are significant at <0.05.

^2^ Intergroup differences calculated via age-adjusted regression model. P-values are significant at <0.0072 (according to Bonferroni correction for multiple testing).

Abbreviations:

PF-ILD: Progressive fibrosing interstitial lung disease; non-PF-ILD: Non-progressive fibrosing interstitial lung disease; TAPSE: Tricuspid annular plane systolic excursion.

Since age differed significantly between groups, an age-adjusted regression model was applied to describe differences in LV strain measurements. When compared to controls, the global longitudinal strain was substantially impaired in PF-ILD patients (-18.3 ± 4.2% vs. -12.7 ± 5.5%, p<0.001) and in non-PF-ILD patients (-18.3 ± 4.2% vs. -14.1 ± 4.6%, p<0.001). The same holds true for the medial septal, medial lateral and basal lateral segmental strain values (Table 4). No differences in strain measurements were observed when PF-ILD patients were compared to the non-PF-ILD group.

Statistically significant correlations were observed between the global longitudinal strain, on the one hand, and hs-CRP, IL-6, TLC and DL_CO_, on the other hand (hs-CRP: Pearson´s r = -0.30, p<0.001; IL-6: Pearson´s r = -0.26, p = 0.007; TLC % predicted: Pearson´s r = 0.22, p = 0.02; DL_CO_ % predicted: Pearson´s r = 0.21, p = 0.02). Except for DL_CO_, significant correlations persisted after age-adjustment (hs-CRP: Pearson´s r = -0.36, p<0.001; IL-6: Pearson´s r = -0.38, p<0.001; TLC % predicted: Pearson´s r = 0.21, p = 0.04).

## Discussion

In the present study, we aimed to determine LV function by speckle tracking-based strain analysis as a function of progressive versus non-progressive fibrosing ILD.

The main findings are as follows: i) ILD is associated with impaired LV contractility. ii) LV dysfunction is accompanied by elevated levels of inflammation markers, and iii) these effects are markedly pronounced in PF-ILD.

Although the diagnostic and therapeutic focus in ILD is primarily pulmonary in nature, a growing body of evidence recognizes ILD as a systemic disease with a substantial comorbidity burden [12,13]. However, available information on comorbid conditions is mainly restricted to IPF [14,15]. In IPF, early mortality not only arises from accelerated respiratory failure, but also from cardiac comorbidities that account for approximately 10% of deaths. These comorbidities primarily comprise congestive heart failure, coronary artery disease, arrhythmias and arterial hypertension [5]. For ILD beyond IPF, data on comorbid conditions are scarce. In a population-based cohort study, Clarson et al. asserted an independent association between ILD–including IPF–and ischaemic heart disease after adjustment for cardiovascular risk factors [16]. Schwarzkopf and colleagues introduced the concept of the `ILD comorbidome’ to visualize comorbidity prevalence and risk of death in ILD [17]. The pathophysiological mechanisms that associate ILD with cardiovascular comorbidities beyond common stressors like smoking is subject of current research. In IPF, oxidative stress caused by intermittent hypoxaemia and complex cytokine derangements may trigger an inflammatory cascade that on the one hand may promote endothelial damage and thereby the atherosclerotic process. On the other, this systemic inflammation is associated with hypercoagulability, leading to formation of microthrombi [5,18]. The transferability of corresponding findings to other ILDs remains less well defined. In the present study, we observed elevated markers of systemic inflammation (leukocytes, hs-CRP, IL-6) in the entire ILD patient group as compared to controls. Intercohortal comparison between alle three groups studied (PF-ILD vs. non-PF-ILD vs. controls) revealed the highest inflammation values in PF-ILD patients, followed by non-PF-ILD patients, with lowest values in controls. Noteworthy, all three here studied inflammation measures were significantly associated with pulmonary function parameters indicating restriction or diffusion limitation. To summarize the observed correlations, the higher the inflammation, the lower TLC, FVC, DL_CO_ and DL_CO_/alveolar volume. The same correlations hold true after excluding all IPF patients (n = 25) from the PF-ILD group. It reinforces the commonalities shared by ILDs of progressive fibrotic phenotype. Moreover, it supports the notion of ILD as a disease accompanied by a pro-inflammatory systemic state that may not be confined to the lungs, but may have downstream systemic consequences.

In keeping with this, we performed speckle tracking analysis to detect subclinical LV dysfunction. Whilst conventional echocardiography LV measures did not offer intercohortal differences, both regional and global longitudinal LV strain was significantly impaired in ILD patients, with PF-ILD patients offering worst strain values und thus worst LV contractility. These observations complement the findings made by Buonauro et al. who assessed biventricular strain in IPF, ILD other than IPF and controls [19]. They identified the global longitudinal LV strain to be altered in IPF patients, but not in patients with ILD of other aetiologies. Contrary to the cited study, we performed not only global, but also regional longitudinal strain assessment by partition of the LV wall into six standard segments. Additionally, we merged all those ILD patients with a progressive fibrotic phenotype (PF-ILD) and compared them to patients with non-progressive fibrosing ILD (non-PF-ILD). As aforesaid, the progressive fibrotic phenotype exhibited the highest degree of LV dysfunction, followed by the non-PF-ILD cohort who still manifested a significantly impaired LV contractility as compared to controls.

Of note, we stated a significant correlation between the global LV strain, on the one hand, and pulmonary function parameters (TLCO, DL_CO_) and markers of systemic inflammation, on the other hand. This observation is consistent with the assumption of persistent low-grade systemic inflammation that may link systemic cardiovascular function to ILD status. Regardless of the heterogeneous nature of its aetiology, congestive heart failure is related to inflammatory activation [20]. In common with other pulmonary disorders like chronic obstructive pulmonary disease, pro-inflammatory events and hypoxia-mediated inflammation in ILD may foster cytokine release and consequently accelerate LV function impairment and heart failure onset [21].

In the current study, age was the only cardiovascular stressor that was not balanced between the PF-ILD and non-PF-ILD cohort, though age did not significantly differ between the entire ILD cohort and controls. In this respect, it should be noted that demographic data on ILD indicate that the progressive fibrotic phenotype appears to be more common in older adults due to a complex interaction of environmental noxae and genetic factors. To evaluate age-confounding on strain analysis results, we applied age-adjusted regression models to describe intergroup differences. After age-adjustment, the observation of a significant distributive difference of global LV strain by intercohortal comparison was upheld. This finding is contrary to the assumption that cardiovascular diseases in ILD may only arise from common risk factors, but points at a multifactorial underlying pathophysiological linkage.

The present study has several methodological limitations including its single centre design that impeded the inclusion of a larger number of ILD patients, in particular with regard to the diverse underlying ILD aetiologies. To overcome this heterogeneity, differentiation between progressive and non-progressive fibrosing ILD was performed, as they share similarities in lung function decline and alveolar dysfunction. Study enrolment was completed in November 2020. In 2022, the term “progressive pulmonary fibrosis” (PPF) was introduced to designate progressive fibrotic disease [22]. Consistent with the INBUILD study criteria to describe PF-ILD, PPF is defined by an interplay of worsening respiratory symptoms, physiological and/or radiologic evidence of disease progression, occurring within 12 months. Remarkably, IPF is defined separately from IPF. We currently did include IPF in the PF-ILD cohort in due consideration of shared similarities regarding pathogenesis and clinical behaviour. As aforementioned, our study´s main results were upheld even after excluding IPF patients from the PF-ILD group. Moreover, assessment of cardiac biomarkers was restricted to NT-proBNP levels. Evaluation of soluble suppression of tumorigenesis-2 (sST2), a member of the interleukin-1 receptor family that reflects cardiovascular stress and fibrosis, and cardiac troponin would have been a valuable adjunct. Finally, we did not aim at establishing causality between ILD and left heart dysfunction, but intended to raise awareness of the need for timely diagnosis of extrapulmonary comorbidities that might be neglected due to the false attribution of common symptoms to the known pulmonary disorder.

In conclusion, ILD was accompanied by LV dysfunction. LV contractility inversely correlated with restrictive ventilatory limitation and inflammation marker levels. Together, these data enhance the concept of ILD as a systemic disease. This holistic approach implies the necessity of periodical active screening for cardiac comorbidities in ILD, especially amongst those with progressive fibrosis.

## Supporting information

S1 Data (XLSX)
